# Heterologous expression, protein folding and antibody recognition of a neurotoxin from the Mexican coral snake *Micrurus laticorallis*

**DOI:** 10.1186/s40409-016-0080-9

**Published:** 2016-09-09

**Authors:** Herlinda Clement, Vianey Flores, Guillermo De la Rosa, Fernando Zamudio, Alejandro Alagon, Gerardo Corzo

**Affiliations:** Departamento de Medicina Molecular y Bioprocesos, Instituto de Biotecnología, Universidad Nacional Autónoma de México (UNAM), Av. Universidad 2001, Cuernavaca, 62210 Morelos Mexico

**Keywords:** *Micrurus laticorallis*, Protein folding, Recombinant, Elapid, Toxin, Protein recognition

## Abstract

**Background:**

The cysteine-rich neurotoxins from elapid venoms are primarily responsible for human and animal envenomation; however, their low concentration in the venom may hamper the production of efficient elapid antivenoms. Therefore, the aim of the present study was to produce fully active elapid neurotoxic immunogens for elapid antivenom production.

**Method:**

Cysteine-rich neurotoxins showed recombinant expression in two strains of *E. coli,* and were purified using affinity chromatography and reverse-phase HPLC (rpHPLC).

**Results:**

The cDNA of the four disulfide-bridged peptide neurotoxin Mlat1 was cloned into a modified expression vector, pQE30, which was transfected into two different *E. coli* strains. The recombinant toxin (HisrMlat1) was found only in inclusion bodies in M15 strain cells, and in both inclusion bodies and cytoplasm in Origami strain cells. The HisrMlat1 from inclusion bodies from M15 cells was solubilized using guanidine hydrochloride, and then purified by rpHPLC. It showed various contiguous fractions having the same molecular mass, indicating that HisrMlat1 was oxidized after cell extraction forming different misfolded disulfide bridge arrangements without biological activity. In vitro folding conditions of the misfolded HisrMlat1 generated a biologically active HisrMlat1. On the other hand, the HisrMlat1 from the cytoplasm from Origami cells was already soluble, and then purified by HPLC. It showed a single fraction with neurotoxic activity; so, no folding steps were needed. The in vitro folded HisrMlat1 from M15 cells and the cytoplasmic soluble HisrMlat1from Origami cells were indistinguishable in their structure and neurotoxicity. Rabbit polyclonal antibodies raised up against biologically active HisrMlat1 recognized the native Mlat1 (nMlat1) from the whole venom of *M. laticorallis*. In addition, HisrMlat1 was recognized by horse polyclonal antibodies obtained from the immunization of elapid species from sub-Saharan Africa.

**Conclusion:**

HisrMlat1 shows increased biological activities compared to the native peptide, and may be used as an immunizing agent in combination with other toxic components such phospholipases type A2 for elapid antivenom production.

## Background

Snake neurotoxins that affect acetylcholine receptor (AChR) have served as a powerful tool to elucidate, both pharmacologically and functionally, different subtypes of AChR. Furthermore, due to their high toxicity, these curare-mimetic toxins represent an important target for the antivenom industry [1, 2]. In the Americas, elapid snakes are represented by coral snakes, which are notable for their red, yellow/white, and black colored banding. In North America, *Micrurus laticollaris* is endemic in Mexico, and its principal habitat is the tropical deciduous forest along the Balsas River in south-central Mexico, which flows through the Mexican states of Puebla, Morelos, Guerrero, and Michoacan, and empties into the Pacific Ocean [3–5]. The venom of this elapid causes neuromuscular blockade in mammalians, which is preceded by a presynaptic effect that facilitates acetylcholine neurotransmitter release [6].

In 2011, the Ministry of Health in Mexico reported 4,024 cases of snakebites (Viperidae and Elapidae) in humans, of which 35 cases were critical and led to human death. The coral snakebites accounted for as much as 5 % of such total cases and fatalities [7, 8]. Mlat1, one of the most neurotoxic molecules of the venom of *M. laticollaris*, binds with high affinity to the mammalian AChR [9]. The primary structure of Mlat1 comprises 60 residues and eight half-cystines that form four disulfide bridges [9]. Since Mlat1 is one of the major venom neurotoxic components that are mainly responsible for *M. laticollaris* envenomation, it is important to be able to generate antibodies that could, eventually, be used to neutralize its effects [10–12]. However, coral snake venoms and their neurotoxins such as Mlat1 are found in minute amounts. Therefore, because of their molecular size, recombinant expression over chemical synthesis seems to be a reliable approach to obtain sufficient amounts of Mlat1 for animal immunization. Consequently, the interest of our research group was to obtain fully active recombinant HisrMlat1 or rMlat1 to employ them as immunogens for further animal immunization. Herein, we report a heterologous expression system for obtaining recombinant HisrMlat1 or rMlat1 with structural and functional characteristics similar to the native one, as well as the antibody recognition proving that the recombinant HisrMlat1 can be used as an immunizing agent.

## Methods

### Bacterial strains, enzymes and plasmids

*E. coli* XL1-Blue was applied for plasmid propagation. The *E. coli* strains M15 and Origami were used for the expression of the toxin-fusion proteins. The plasmids TOPO 2.1 (Invitrogen, USA) and pQE30 (Qiagen, Germany) were employed for cloning and production of the fusion proteins with a 6His-tag, respectively. Restriction enzymes BamHI, PstI, *Taq* polymerase, Factor Xa and T4 DNA ligase were purchased from New England Biolabs (USA).

### Gene cloning

Based on the information obtained from direct peptide sequencing of Mlat1, a specific oligonucleotide was designed and used for the PCR reaction using as a template the cDNA material from a cDNA library created with *M. laticollaris* venom gland. The PCR reaction was performed in 1X Taq DNA polymerase with ThermoPol (New England Biolabs, USA), 200 μM dNTPs, 0.25 μM forward primer Mlatfw (5-AGG ATA TGT TAC AAC CAA CAG - 3′); 0.25 μM reverse Mlatrv primer (5′-ACC GTT GCA TTT GTC TGA TGT -3′) and two units of Taq DNA polymerase in a final volume of 50 μL in a Applied Biosystems Gene Amp 9700 instrument. The Taq DNA polymerase was added and the mixture was then incubated at 94 °C for 3 min for one cycle. After the initial cycle, the mixture was incubated at 94 °C for 30 s, 58 °C for 2 min and 72 °C for 2 min per 30 cycles, followed by a final 7 min step at 72 °C. PCR products were purified using a High Pure PCR Product Purification Kit (Roche, Switzerland) following the manufacturer’s instructions, and then ligated into a Topo 2.1. The ligation reaction was used to transform competent *E. coli* XL1-Blue cells. Positive clones were sequenced from both ends using the Thermo Sequenase Radiolabeled Terminator Cycle Sequencing Kit (Amersham, USA).

### Plasmid construction

**Fig. 1:**
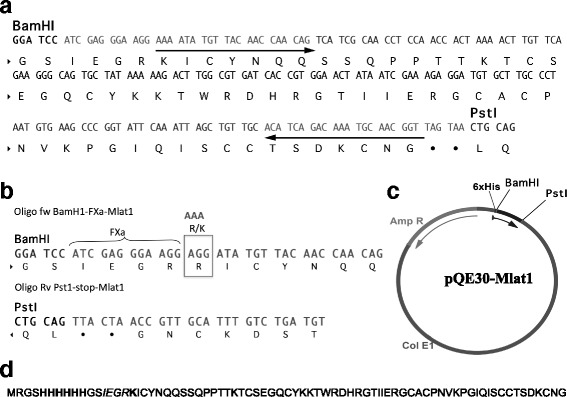
Gene construction of the plasmid carrying HisrMlat1. **a** The nucleotide and amino acid sequence of HisrMlat1. **b** The nucleotide and amino acid sequence of HisrMlat1 after modification of the Arg N-terminal. **c** Scheme of the expression vector pQE30XaHisrMlat1. The His-tag is part of the 5′-nucleotide sequence of pQE30, in such a manner that the sequence to be expressed is the one shown, in which the cleavage site for the restriction enzyme is indicated (BamHI and PstI). **d** Amino acid sequence of HisrMlat1. The His-tag is in bold, the FXa cleavage site is in italics and the modification of the residue Lys is in bold

The DNA fragment encoding the Mlat1 sequence, preceded by a Factor Xa recognition site, was amplified by PCR from a cDNA clone obtained from the library previously described. Thus, the plasmid contained the 6His-tag, the sequence coding for the amino acids recognized by the protease Factor Xa (FXa) and the Mlat1 gene. Since the last residue of the recognition site for FXa is arginine (IEGR) and the first residue of the N-terminal Mlat1 is also an Arginine (RIC…), the FXa can cleave after the second Arginine (IEGRR/IC) leaving a truncated N-terminal rMlat1. Therefore, the first Arginine in Mlat1 was replaced by a basic amino acid Lysine to avoid this putative problem. Therefore, the plasmid construction was denominated pQE30XaMlat1, and its product was abbreviated HisrMlat1 (Fig. **1**).

Appropriate oligonucleotides were designed for introducing the enzymatic restriction sites *Bam*HI and *Pst*I for directional insertion into pQE-30. The PCR-amplified product was cloned into TOPO 2.1, as well as for introducing the residue Lys instead of Arg at the N-terminal of Mlat1. The PCR product was subcloned using standard methods. The PCR amplifications were carried out using the following oligonucleotides: Oligo fw Bam-Fxa-Mlat1 that corresponds to 5′-GGA TCC ATC GAG GGA AGG AAA ATA TGT TAC AAC CAA CAG-3′, and Oligo Rv Pst1-stop-Mlat1 that corresponds to 5′-CTG CAG TTA CTA ACC GTT GCA TTT GTC TGA TGT-3′. The PCR conditions were: the Taq DNA polymerase was added and the mixture was then incubated at 94 °C for 3 min for one cycle. After the initial cycle, the mixture was incubated at 94 °C for 30 s, 58 °C for 2 min and 72 °C for 2 min per 30 cycles, followed by a final seven-minute step at 72 °C. The PCR product was cloned in TOPO 2.1.

The corresponding plasmids were digested with the corresponding restriction enzyme EcoRI at 37 °C for 1 h and purified using a High Pure PCR Product Purification Kit (Roche, Switzerland) following the manufacturer’s instructions, before ligation. The ligation reaction (20 μL) was carried out with T4 DNA ligase with a six-fold insert excess over plasmid (pQE30) for 16 h at 16 °C. Ten microliters of the ligation reaction was used to transfect competent cells of either *E. coli* M15 or Origami. Positive clones with the expected insert were grown in Luria-Bertani broth (LB) supplemeted with ampicillin and kanamycin. The plasmids of positive colonies were purified using the High Pure Plasmid Isolation Kit (Roche, Switzerland). Plasmid constructs were verified by sequencing from both sites, the insert boundaries to confirm the reading frame and conservation of restriction sites. M15 strains were transfected with the corresponding plasmid for 2 min at 42 °C, followed by 5 min in ice and 30 min recovery at 37 °C in LB medium. Plates of LB contained 100 and 25 μg/mL of ampicillin and kanamycin, respectively.

### Expression and purification of HisrMlat1

*E. coli* strain M15 or strain Origami expressing the plasmid pQE30XaMlat1 was grown in LB medium. After the absorbance at 600 nm reached 0.6 absorption units, the cultures were induced with 0.1 mM IPTG (isopropyl-β-D-thiogalactopyranoside) for 6 h at 30 °C. Cells were harvested by centrifugation (8,000 rpm for 20 min at 4 °C) using a Beckman centrifuge model J2-21, then recovered and lysed with a BugBuster^®^ protein extraction reagent (Merck KGaA, Germany). This material was centrifuged again (8,000 rpm for 20 min at 4 °C) and the supernatant and the insoluble fraction (inclusion bodies) were recovered. The supernatant was dissolved in a 0.05 M Tris-base buffer pH 8.0, and the inclusion bodies were dissolved in 6 M GndHCl in a 0.05 M Tris-base buffer pH 8.0.

Purification of the HisrMlat1, from either supernatant or inclusion bodies, using Ni-NTA (Ni-nitrilotriacetic acid) affinity column chromatography was performed according to the instructions of the manufacturer (Qiagen, USA). Finally, the recombinant product was eluted with their respective buffers containing 250 mM imidazole (pH 8.0). Imidazole was eliminated by a second purification step under reverse-phase HPLC (rpHPLC) system using an analytic C_18_ rpHPLC column (Vydac 218TP 4.6 x 250 mm, Hesperia, USA) and an elution gradient from 20 % to 60 % solvent B for 40 min. Solvent A was 0.1 % trifluoroacetic acid (TFA) in water and solvent B was 0.1 % TFA in acetonitrile.

The eluted HisrMlat1 was vacuum dried. The HisrMlat1 product from inclusion bodies was reduced using 50 mM DTT in 0.05 M Tris-base buffer, pH 8.0, and was allowed to fold under controlled conditions using 2 M GndHCl in 0.05 M Tris-base buffer, pH 8.0, containing a redox pair. The identity of the toxin was confirmed by both automatic Edman degradation and mass spectrometry analysis using a Finnigan LCQ^DUO^ ion trap mass spectrometer (USA). These techniques are currently used in the laboratory and are reported elsewhere [13].

### Polyclonal immunization protocol

New Zealand white rabbits (3–4 kg, NZW) were used for immunization with HisrMlat1, by subcutaneous route, starting with 2 μg and ending with 60 μg peptide/rabbit. The first immunization was performed in 500 μL PBS plus 500 μL complete Freund’s adjuvant (CFA). Rabbits were boosted 7, 14, 21, 28, 35, 42, 49, 56, 63, 70 and 77 days later, with 2, 5, 10, 10, 20, 50, 50, 50, 50, 50 and 50 μg of HisrMlat1, respectively, in incomplete Freund’s adjuvant (IFA) and then alternating IFA and aluminum hydroxide (Alum) until the last immunization. Rabbits were bled on the first day (pre-immune) and after immunization. Each immune serum was tested using ELISA for the presence of antibodies against native Mlat1.

### Secondary structure

The circular dichroism spectra (CD) of native Mlat1 (nMlat1) and HisrMlat1 were recorded in a Jasco J-710 spectropolarimeter (Jasco, Japan), from 190 to 260 nm, in 60 % trifluoroethanol (TFE), at room temperature, with a 1 mm-path quartz cell. Data were collected every 0.5 nm at the velocity of 20 nm/min. The respective concentrations of nMlat1 and HisrMlat1 were 0.4 and 0.6 mg/mL. The CD values correspond to the mean of two recordings. Percentages of secondary structure content were calculated from the spectra using the K2D3 web server [14].

### Biological activity

The biological activity of HisrMlat1 in vivo was evaluated via a mouse model according to the guidelines of our Institute’s Committee for Animal Welfare, keeping the number of animals to the minimum required to validate the experiments. Male mice (CD-1, 20 g body weight) were tested by intravenous injection. Pure recombinant peptides HisrMlat1 or rMlat1 were diluted up to 500 μL with PBS and injected with a 1 mL syringe.

## Results and Discussion

### cDNA cloning and recombinant expression

As described in the Material and Methods section, a gene coding for Mlat1 toxin was obtained from a cDNA library (Fig. 1a) [9]. The Mlat1 gene was modified to include a six-histidine segment followed by four amino acids corresponding to a cleavage site of the enzyme Factor Xa, and a variation in the first residue of the mature protein (R/K) (Fig. 1b and c). After the gene was amplified, the product with the expected size (~230 bp) was purified via the High Pure Plasmid Isolation Kit (Roche, Germany) cloned into PCR®2.1-TOPO vector (Invitrogen, USA) and its sequence was verified and the plasmid pQ30XaMlat1 was constructed (Fig. 1c). This plasmid was confirmed to contain the DNA sequence to code a segment of six histidines followed by four amino acids corresponding to cleavage site of the enzyme Factor Xa, and the 60 amino acid residues corresponding to the mature toxin isolated from *M. laticorallis* (Fig. 1d).

### Purification, in vitro folding and enzymatic cleavage

**Fig. 2:**
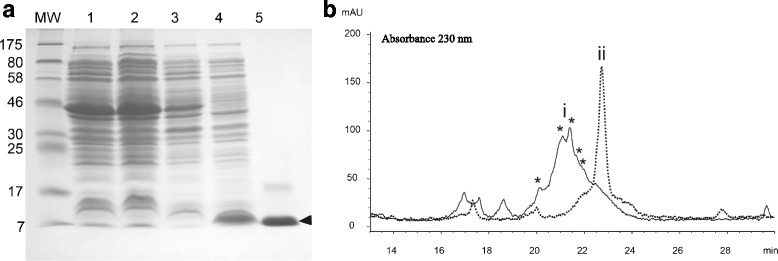
SDS-PAGE and HPLC separation of HisrMlat1 from M15 strain cells. **a** SDS-PAGE. Left lane shows the molecular weight markers (kDa). Lane 1: the cell protein content that has the pQE30 vector without the HisrMlat1 gene and without IPTG induction. Lane 2: the cell protein content that has the pQE30 vector without the HisrMlat1 gene after 0.1 mM IPTG induction. Lane 3: the cell protein content that has the pQE30 vector with the HisrMlat1 gene and without IPTG induction. Lane 4: the cell protein content that has the pQE30 vector with the HisrMlat1 gene and with 0.1 mM IPTG induction. Lane 5: the protein after purification by affinity column (upper bands are oligomers of HisrMlat1). The protein band corresponding to HisrMlat1 is indicated by an arrow. **b** rpHPLC. The product obtained from the affinity column was directly loaded into the C_18_ reverse column (approx. 100 μg of protein). The fractions labeled with an asterisk had the molecular mass expected for the recombinant HisrMlat1 (label i). None of the fractions were lethal to mice. The pooled multiple oxidized fractions (100 μg of protein, label i) were reduced with DTT, and loaded into the same rpHPLC. Superimposed on this chromatogram is the profile of the reduced HisrMlat1 (label ii)

*E. coli*, either strain M15 or strain Origami, expressed the His-tagged FXa recombinant HisrMlat1 peptide. The cells obtained after induction with IPTG and decanted by centrifugation were resuspended and ruptured with the BugBuster^®^ protein extraction reagent as described in Material and Methods. Figure 2a shows the SDS-PAGE of proteins expressed in *E. coli* strain M15. *E. coli* cells that neither had the HisrMlat1 gene nor were induced by IPTG expressed the recombinant HisrMlat1 (lanes 1 to 3).

After IPTG induction of the vector in *E. coli* strain M15, a presence of a protein band with the expected molecular weight of HisrMlat1 was obtained (lane 4). Furthermore, the recombinant HisrMlat1 from inclusion bodies was purified using affinity chromatography (lane 5). Subsequently, the imidazole eluate from the affinity chromatography separation (sample observed in lane 5 of Fig. 2a) was directly loaded into a C_18_ column for rpHPLC purification (Fig. 2B). Interestingly, all the chromatographic peaks marked with an asterisk had the expected molecular mass of the recombinant HisrMlat1 peptide (8,499.6Da) suggesting multiple oxidized forms (Fig. 2b-i, solid lines). Those oxidized isoforms were probably generated during extraction of inclusion bodies that resulted in the oxidation of thiol groups, which provide structure to scramble disulfide bridges. The formation of multiple oxidized isoforms of cysteine-rich neurotoxins have been observed previously [15]. Therefore, the cystines of those oxidized isoforms were chemically reduced. Figure 2B-ii (discontinued lines) shows the results of the rpHPLC separation after chemical reduction of a pool of HisrMlat1 fractions oxidized by DTT. A single fraction of 8,507.6 Da was observed; that is, 8 Da more than the expected mass because of the reduction of four cystines. The reduced HisrMlat1 was folded by following different in vitro conditions to yield several oxidized fractions (data not shown). Folding conditions using 2 M GndHCl in 0.05 M Tris-base buffer, pH 8.0, containing the redox pair CysCys/Cys, bring in an active HisrMlat1.

**Fig. 3:**
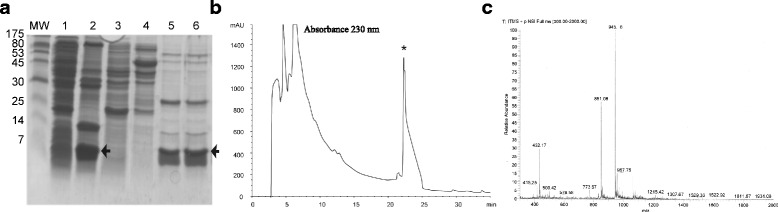
SDS-PAGE and HPLC separation of HisrMlat1 from Origami strain cells. **a** SDS-PAGE. Left lane shows the molecular weight markers (kDa). Lane 1: the protein extract from cytoplasm. Lane 2: the protein content in the cytoplasmic soluble fraction (an arrow indicates the relative molecular size of the expected HisrMlat1). Lanes 3 and 4: the proteins that were not bound to the Ni-NTA column. Lanes 5 and 6: the eluates from the Ni-NTA after 250 mM imidazole. The protein bands corresponding to HisrMlat1 are indicated by an arrow. **b** rpHPLC. The product obtained from the affinity column was directly loaded into the C_18_ reverse column (approx. 500 μg of protein). The fraction labelled with an asterisk had the molecular mass expected for the recombinant HisrMlat1. This fraction was lethal to mice. **c** Mass spectrometry. HisrMlat1 from either M15 or Origami strain cells has the same molecular mass, namely 8,499.6. The main m/z ions are shown [945.8]^+9^, [851.08]^+10^, [773.67]^+11^

On the other hand, after IPTG induction of the vector in *E. coli* strain Origami, the SDS-PAGE of proteins obtained from the cytoplasm of the disrupted cells showed several proteins of different molecular sizes (Fig. 3a, lane 1). However, in the soluble fraction was noted the presence of a protein band with the approximate expected molecular size of HisrMlat1 (lane 2, arrow). Therefore, protein fractions obtained from the cytoplasm or from inclusion bodies were separated by means of affinity chromatography. Lanes 3 and 4 represent the first and second eluates without imidazole, from the cytoplasmic soluble fraction, showing that His-tagged Mlat1 (HisrMlat1) was kept within the Ni-NTA column. Lanes 5 and 6 represent the eluates with 250 mM imidazole indicating the recuperation of the HisrMlat1 from the Ni-NTA column. Interestingly, the rpHPLC separation of the soluble fraction yielded a single oxidized fraction (Fig. 3b) with the expected molecular mass (Fig. 3c).

### Evidence of similar structural characteristics

**Fig. 4:**
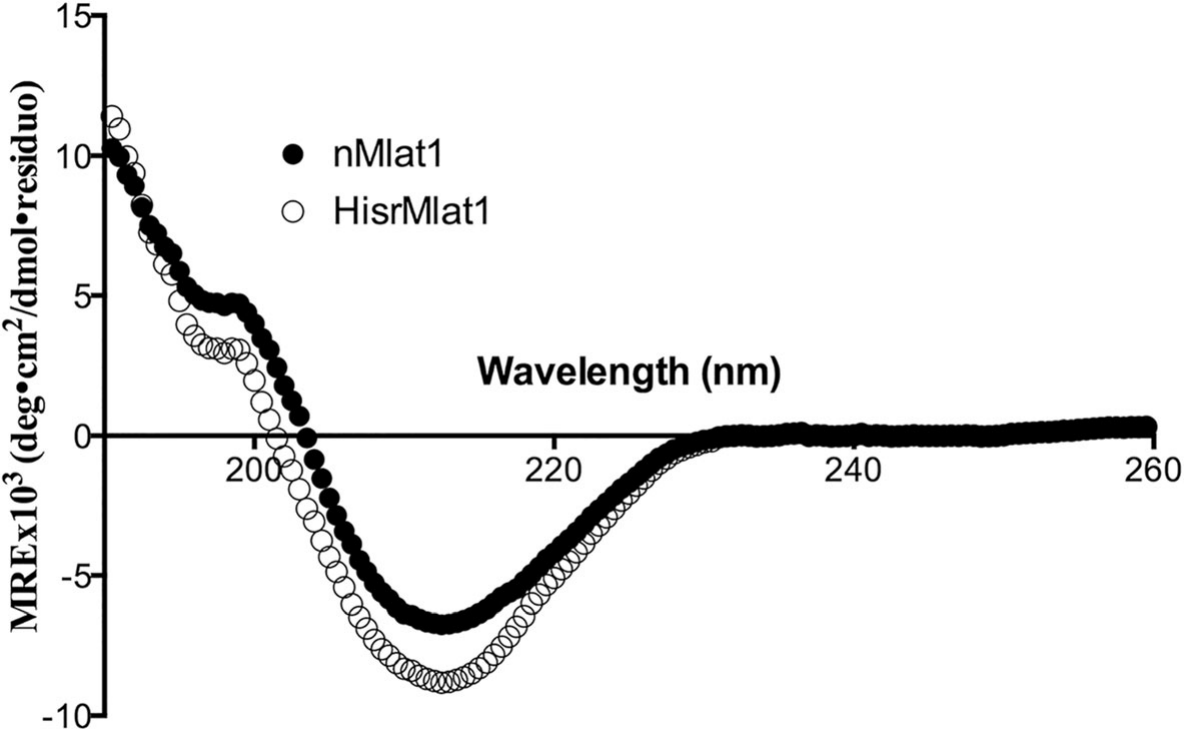
Circular dichroism of native nMlat1 and recombinant HisrMlat1. Secondary structural analysis showed 4.7 and 9.7 % of α helix and 35.3 and 30.0 % of β strand for nMlat1 and HisrMlat1, respectively

Since the recombinant HisrMlat1 obtained from either M15 or Origami cells had similar retention times and biological activity indicating similar structural identities, they were combined for further analysis of secondary structure and biological activity. The secondary structure of HisrMlat1 was analyzed using circular dichroism (CD). The CD spectra of the native and recombinant HisrMlat1 showed low absorption for the α-helix secondary structure, and a relative high content in β-strands (Fig. 4). According to the CD deconvolution program of Louis-Jeune et al. [14], the respective secondary structure contents were 4.7 and 9.7 % of α helix and 35.3 and 30.0 % of β strand for nMlat1 and HisrMlat1. Both nMlat1 and HisrMlat1 adopt the typical β-strand motif of the structurally characterized α-neurotoxins from elapids, which are exemplified by negative ellipticities at 210–215 nm [16]. Herein, Mlat1 resembles the spectrum of long African elapid neurotoxins with a minimum at 210 nm, and a reduced positive band at 195 nm instead of the large positive band at 195 nm of the short African elapid neurotoxins [16].

### Biological activity and antibody recognition

**Fig. 5:**
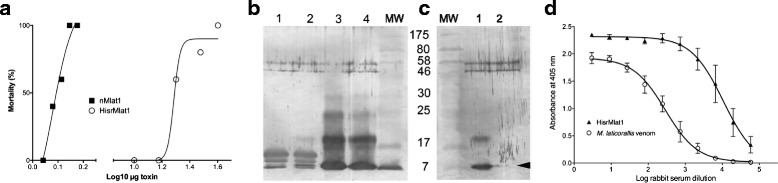
Biological activity of native Mlat1 and HisrMlat1. **a** Dose-response of mice to intravenous injection of native Mlat1 and HisrMlat1. **b** Western-blot of *Micrurus laticorallis* venom (lanes 1 and 2) and HisrMlat1 (lanes 3 and 4) that were recognized using serum of rabbits immunized with recombinant HisrMlat1. Lane MW corresponds to the molecular weight markers (kDa). **c** Western-blot of HisrMlat1 (lane 1) and nMlat1 (lane 2) that were recognized using serum of horses immunized with sub-Saharan African elapid venoms. Lane MW corresponds to the molecular weight markers (kDa). **d** ELISA detection of *M. laticollaris* venom and HisrMlat1 using sera of rabbits immunized with HisrMlat1

The LD_50_ of the native Mlat1 has been reported to be 0.064 mg/kg of mouse weight when injected intravenously [9]. Therefore, groups of five mice were injected intravenously with HisrMlat1. The LD_50_ for HisrMlat1 was observed at 0.95 mg/kg mice (Fig. 5a); that is, 16 times less toxic than the native peptide, which might be caused by the extra 16 amino acids at its N-terminal. Similarly, the recombinant HisrCssII, a four-disulfide-bridged neurotoxin from the venom of the scorpion *Centruroides suffuses suffusus*, had lower toxicity than that of the native CssII. It was proven that the 16-merfusion protein added to the N-terminal of CssII decreased its toxicity when compared to the native protein. Correspondingly, the native CssII had 15-fold higher binding affinity than that of the HisrCssII [15]. This result implies that the 16-mer N-terminal fusion protein hampers the receptor recognition in those two recombinant neurotoxins, HisrMlat1 and HisrCssII.

Nevertheless, rabbit polyclonal antibodies raised against HisrMlat1 recognize the native Mlat1, which suggests that the recombinant Mlat1 could be used for venom neutralization (Fig.5b). Furthermore, plasma of horses immunized with sub-Saharan African elapids (*Dendroapsis polylepis*, *D. angusticeps*, *D. viridis*, *D. jamesoni*, *Naja nigricollis*, *N. melanoleuca*, *N. nivea*, *N. haje* and *N. katensis*) recognized the recombinant and native Mlat1 suggesting a well-structured recombinant neurotoxin (Fig. 5c). Unfortunately, the rabbit antibodies produced against HisrMlat1 did not neutralize the *M. laticorallis* venom and it had low titers (EC_50_ = 290) compared to the recognition of HisrMlat1 (EC_50_ = 10,703) (Fig. 5d). The absence of neutralization may be attributable not only to the low titers, but also to the presence of other lethal components in the whole coral snake venom, which contains phospholipase A2 that also exerts strong toxicity on mammalian acetylcholine receptors. Therefore, efforts to express recombinant elapid neurotoxins and phospholipase A2 could yield a true elapid antivenom.

In this work, we explore only the recognition of rabbit and horse sera to Mlat1 from *M. laticollaris*. The rabbit serum was originated by immunization with the recombinant HisrMlat1, whereas the horse serum was generated by immunization with sub-Saharan African elapid venoms. Therefore, efforts to express recombinant elapid neurotoxins and phospholipases A2, to improve immunization protocols, and to use the proper animals for immunization must be explored for achieving universal elapid anti-venoms.

Even though these data point towards the use of universal elapid antivenoms, recently, Tanaka et al. [17] used several *Micrurus* venoms to obtain serum by immunizing three different animals, specifically rabbits, mice and horses. Although the serum from those three animal species exhibited the same immunogenicity pattern, and that each specific serum presented cross-reactivity when analyzed by ELISA and Western blot techniques, the results were poorly correlated with the neutralizing potential of the antivenom. Only the horse antivenom neutralized the coral snake venom [17]. Therefore, producing novel and more efficient *Micrurus* sp. antivenoms using recombinant elapid neurotoxins is not a simple task because other proteins such as phospholipases A2 must be complemented to produce immunizing agents.

## Conclusion

This report shows the expression, folding, secondary structure, and biological activity as well as rabbit antibody recognition of the four-disulfide-bridged recombinant neurotoxin HisrMlat1. As mentioned in the introductory section, it is an important contribution due to the fact that the literature has not reported in vivo toxicity of recombinant coral snake neurotoxins possibly because of wrongly folded toxins. This report shows that the active recombinant coral snake neurotoxin could be obtained from either inclusion bodies or the cytoplasmic fraction depending on the bacterial cells used. These results also open up the production of site-directed mutants to study the structural-functional relationship for coral snake neurotoxins with acetylcholine receptors as well as for further research towards coral snake antivenom production. Furthermore, the fact that the serum from horses immunized with sub-Saharan African elapid venoms recognized the recombinant HisrMlat1 provides a pathway for recombinant expression of this type of three-finger neurotoxins as universal immunizing agents for developing antivenoms for neurotoxic elapids. Finally, it is important to point out that elapid neurotoxins are one of the toxic components of the whole venom and other components such phospholipase A2 are needed as immunogens to make reliable antivenoms.

## Abbreviations

TFA: Trifluoroacetic acid
ESI: Electro-spray ionization
rpHPLC: Reverse-phase HPLC
Mlat1: Toxin 1 from the elapid *Micrurus laticollaris*
